# Comparing porous tantalum fusion implants and iliac crest bone grafts for spondylodesis of thoracolumbar burst fractures: Prospectice Cohort study

**DOI:** 10.1038/s41598-021-96400-w

**Published:** 2021-08-31

**Authors:** Martin C. Jordan, Hendrik Jansen, Rainer H. Meffert, Timo M. Heintel

**Affiliations:** Department of Orthopaedic Trauma, Hand, Plastic and Reconstructive Surgery, University Hospital Würzburg, Julius-Maximilians-University, Oberdürrbacher Str. 6, 97080 Würzburg, Germany

**Keywords:** Fracture repair, Musculoskeletal system

## Abstract

The aim of this study was to compare two different techniques of performing one-level spondylodesis for thoracolumbar burst fractures using either an autologous iliac crest bone graft (ICBG) or a porous tantalum fusion implant (PTFI). In a prospective nonrandomized study, 44 patients (20 women, 24 men; average age 43.1 ± 13.2 years) suffering from severe thoracolumbar burst fractures were treated with combined anterior–posterior stabilization. An ICBG was used in 21 cases, and a PTFI was used in the other 23 cases. A two-year clinical and radiographic follow-up was carried out. There were no statistically significant differences in age, sex, localization/classification of the fracture, or visual analog scale (VAS) before injury between the two groups. All 44 patients were followed up for an average period of 533 days (range 173–1567). The sagittal spinal profile was restored by an average of 11.1° (ICBG) vs. 14.3° (PTFI) (monosegmental Cobb angle). Loss of correction until the last follow-up tended to be higher in the patients treated with ICBG than in those treated with PTFI (mean: 2.8° vs. 1.6°). Furthermore, significantly better restoration of the sagittal profile was obtained with the PTFI than with the iliac bone graft at the long-term follow-up (mean: ICBG 7.8°, PTFI 12.3°; *p* < 0.005). Short-segment posterior instrumentation combined with anterior one-level spondylodesis using either an ICBG or a PTFI resulted in sufficient correction of posttraumatic segmental kyphosis. PTFI might be a good alternative for autologous bone grafting and prevent donor site morbidities.

## Introduction

**Figure 1:**
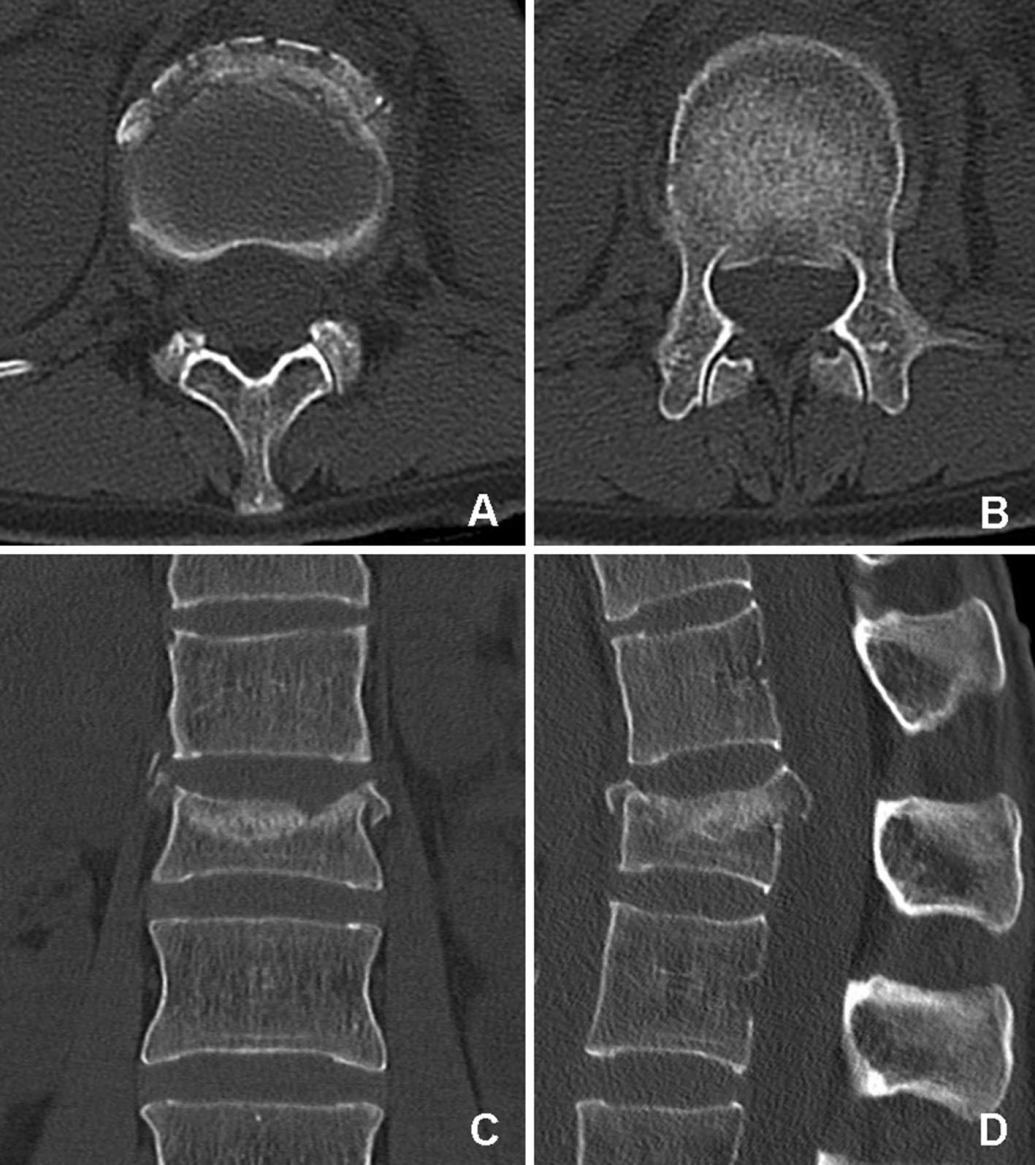
Preoperative CT of a 42-year-old woman with a superior incomplete burst fracture (Magerl et al.^19^ A3.1.1, AOSpine A3^1^, load sharing classification score 7^3^) of L1 sustained in a car accident. CT scans showing representative axial (**A** and **B**), coronal (**C**) and sagittal (**D**) slices of the thoracolumbar junction. *The upper half of the vertebral* body was partially comminuted with centrifugal extrusion of fragments. Fragments of the posterior wall are retropulsed into the spinal canal.

The optimal treatment for traumatic burst fractures of the thoracolumbar spine remains controversial (Fig. 1). In the absence of conclusive studies, the management of thoracolumbar injuries is based on expert opinions rather than evidence-based medicine. Furthermore, the lack of evidence to determine which treatment is superior has led to substantial regional treatment variability^1,2^.

A major challenge after posterior correction and instrumentation in the treatment of severe thoracolumbar burst fractures is supporting the anterior spinal column. McCormack et al.^3^ proposed a load sharing classification system to help identify fractures that require anterior stabilization. With this system, the fractured vertebra is scored on the basis of the amount of comminution, fracture apposition, and kyphosis. Fractures with a score of 7 or more points require anterior stabilization, whereas those with a score of 6 or fewer points can be treated with an isolated posterior approach. Nonoperative therapy also serves as an alternative. Endplate comminution is strongly associated with disc degeneration, which usually occurs at the level adjacent to the fractured endplate of thoracolumbar burst fractures and leads to gradual loss of correction, hardware breakage, and poor radiological and clinical results^3–5^. Due to severe biomechanical instability in these types of fractures, an increasing number of spine surgeons favor a combined anterior and posterior procedure using anterolateral interbody fusion and short pedicle screw fixation^3,6–11^. The advantages of combined surgical approaches are ideal restoration of the vertebral body height and rigid stabilization in all 3 planes of spinal motion. On the other hand, posterior-anterior stabilization is technically more difficult, is more costly and can lead to more morbidities than posterior-only stabilization^12^.

Autologous tricortical iliac crest bone grafts (ICBGs) are considered the gold standard graft material for fusion in anteroposteriorly stabilized thoracolumbar fractures, but there are several important disadvantages of using autologous bone, including a risk of donor-site morbidities and a relatively high frequency of nonunion^13–15^. Additionally, the availability of autologous bone grafts is limited and may be insufficient, particularly in patients with spondylodesis spanning multiple segments^16^. Therefore, synthetic interbody spinal fusion devices are becoming increasingly important. Highly porous tantalum implants are among the latest developments in this field. Tantalum is a highly biocompatible and corrosion-resistant metal. Porous tantalum has a low modulus of elasticity (2.5–3.9 gigapascal (GPa)) that is more similar to that of cancellous bone than that of titanium (106–115 GPa)^17,18^. The implant is strong enough to accommodate loads imposed by adjacent vertebrae and is porous enough to facilitate tissue ingrowth and bony fusion^17,18^. The porosity is provided by a plurality of randomly sized, substantially interconnected voids disposed throughout the body.

The aim of this prospective observational study was to compare two different techniques for anterior one-level spondylodesis in thoracolumbar burst fractures using either an ICBG or PTFI. The techniques may differ in their operative risks, complications, outcomes, and resource use.

## Methods

### Inclusion and exclusion criteria

Strict inclusion and exclusion criteria (Table 1) were chosen to identify a relatively homogeneous study population.

**Table 1:** Inclusion and exclusion criteria.

**Inclusion criteria** Recent fracture (< 6 weeks) of the thoracolumbar spine Fracture type A3.1.1/AOSpine A3 (superior incomplete burst fracture) or A3.2.1/AOSpine A4 (superior burst-split fracture) according to Magerl et al.^19^/Vaccaro et al.^1^ without dislocation (< 2 mm) of the sagittally split lower part of the vertebral body Amount of comminution/involvement of the vertebral body ranging 30–60%^3^ Load sharing score of 6 or more points according to the method described by McCormack et al.^3^ Patient age of 20 to 65 years Prospectively collected data **Exclusion criteria** Pathologic or osteoporotic fractures Serial spine fractures or multilevel noncontiguous spine fractures Acute traumatic spinal cord injuries (Frankel Type A-C)^20^	

During a period of 4 years (January 2009–December 2012), 44 nonrandomized patients (20 women, 24 men) with an average age of 43.1 ± 13.2 years (range 20–65 years) were included in the study (Table 2). All patients gave their informed consent before participation. For this study, we received institutional review board approval in 2008 (approval no. 38/08) from the JMU university.

**Table 2:** Patient demographics. Iliac crest bone graft (ICBG), porous tantalum fusion implant (PTFI).

Case No	Sex	Age (years)	Smoker/nonsmoker	Localization	Fracture type (Magerl et al./AOSpine^1,19^	Load Sharing Classification Score^3^	ICBG/PTFI
1	Male	42	Nonsmoker	L1	A3.2.1/A4	7	ICBG
2	Female	24	Smoker	T12	A3.2.1/A4	7	ICBG
3	Male	37	Smoker	L1	A3.1.1/A3	7	ICBG
4	Female	34	Nonsmoker	L1	A3.1.1/A3	7	PTFI
5	Female	25	Nonsmoker	T12	A3.2.1/A4	7	PTFI
6	Male	56	Nonsmoker	L2	A3.2.1/A4	7	PTFI
7	Male	53	Nonsmoker	L3	A3.1.1/A3	6	ICBG
8	Male	47	Nonsmoker	L1	A3.1.1/A3	7	ICBG
9	Female	62	Nonsmoker	L2	A3.1.1/A3	6	ICBG
10	Male	45	Nonsmoker	T12	A3.1.1/A3	6	PTFI
11	Female	29	Smoker	L2	A3.2.1/A4	7	PTFI
12	Female	36	Smoker	L1	A3.2.1/A4	7	ICBG
13	Female	54	Nonsmoker	L1	A3.1.1/A3	6	ICBG
14	Female	51	Nonsmoker	L1	A3.2.1/A4	7	PTFI
15	Male	25	Nonsmoker	L3	A3.1.1/A3	6	PTFI
16	Male	41	Nonsmoker	L2	A3.1.3/A3	6	ICBG
17	Female	38	Nonsmoker	L1	A3.2.1/A4	7	ICBG
18	Male	51	Nonsmoker	L2	A3.2.1/A4	7	PTFI
19	Female	22	Nonsmoker	T12	A3.1.1/A3	6	ICBG
20	Male	43	Nonsmoker	L1	A3.2.1/A4	7	ICBG
21	Male	53	Nonsmoker	L2	A3.2.1/A4	7	ICBG
22	Male	65	Nonsmoker	L1	A3.2.1/A4	7	PTFI
23	Male	45	Nonsmoker	L3	A3.1.1/A3	6	ICBG
24	Female	65	Nonsmoker	L1	A3.1.1/A3	6	ICBG
25	Female	34	Nonsmoker	L1	A3.2.1/A4	7	ICBG
26	Male	61	Smoker	L1	A3.2.1/A4	7	PTFI
27	Female	25	Smoker	L1	A3.1.1/A3	6	PTFI
28	Male	63	Smoker	L1	A3.2.1/A4	7	ICBG
29	Male	26	Smoker	L1	A3.2.1/A4	7	ICBG
30	Female	22	Nonsmoker	L2	A3.1.1/A3	6	PTFI
31	Male	30	Nonsmoker	L4	A3.1.1/A3	6	ICBG
32	Female	42	Nonsmoker	L1	A3.1.1/A3	6	ICBG
33	Female	51	Nonsmoker	L1	A3.1.1/A3	6	PTFI
34	Male	57	Nonsmoker	T12	A3.1.1/A3	6	PTFI
35	Female	35	Nonsmoker	L1	A3.2.1/A4	7	PTFI
36	Male	50	Nonsmoker	L1	A3.2.1/A4	7	PTFI
37	Male	20	Nonsmoker	L1	A3.2.1/A4	7	PTFI
38	Male	47	Nonsmoker	L1	A3.2.1/A4	7	PTFI
39	Male	55	Smoker	L1	A3.2.1/A4	7	PTFI
40	Male	38	Nonsmoker	L1	A3.1.1/A3	6	PTFI
41	Female	58	Nonsmoker	L1	A3.1.1/A3	6	PTFI
42	Female	59	Nonsmoker	L2	A3.1.1/A3	6	ICBG
43	Male	38	Nonsmoker	L1	A3.1.1/A3	6	PTFI
44	Female	39	Smoker	T12	A3.1.1/A3	6	PTFI

### Surgical technique

**Figure 2:**
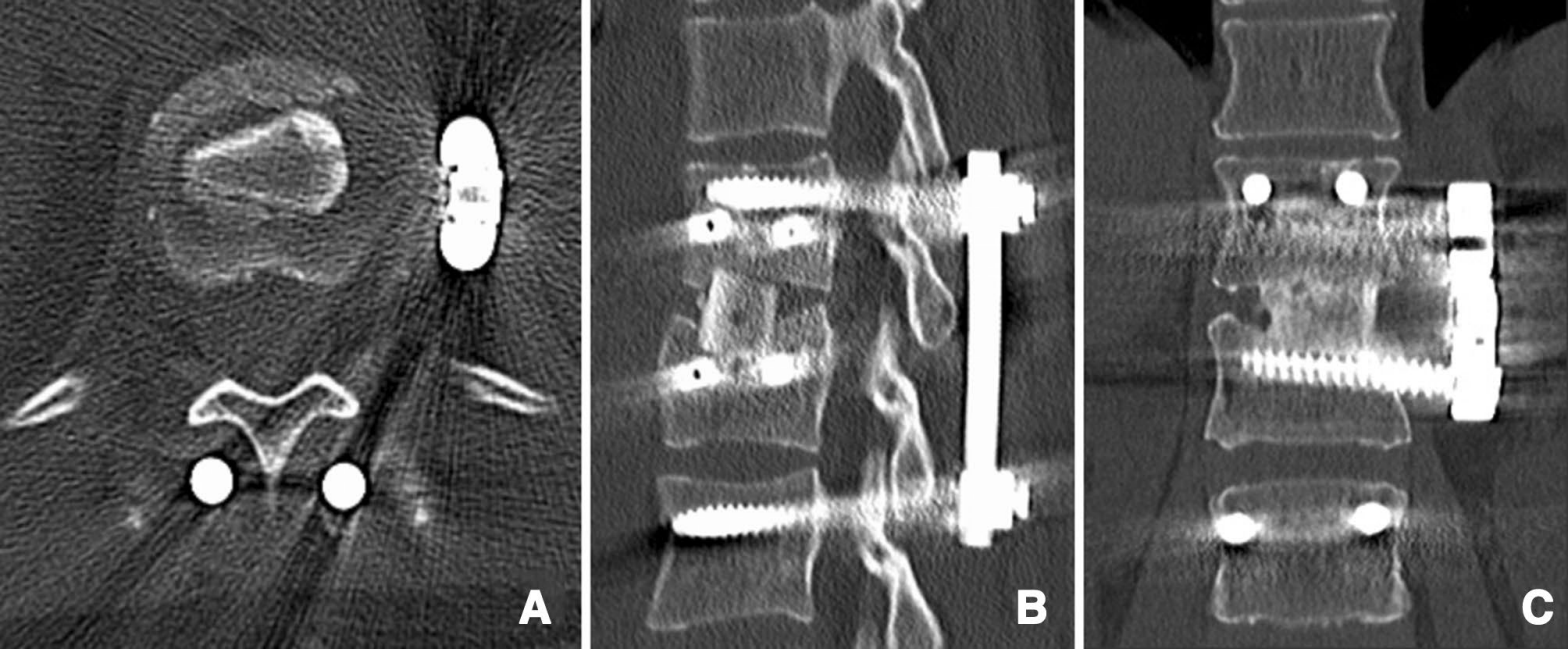
Postoperative CT of a 38-year-old woman with a superior burst-split fracture (Magerl et al.^19^ A3.2.1, AOSpine A4^1^) of L1 treated by short segment posterior instrumentation and anterior one-level spondylodesis using an iliac crest bone graft (ICBG). Representative axial (**A**), sagittal (**B**) and coronal (**C**) slices.

**Figure 3:**
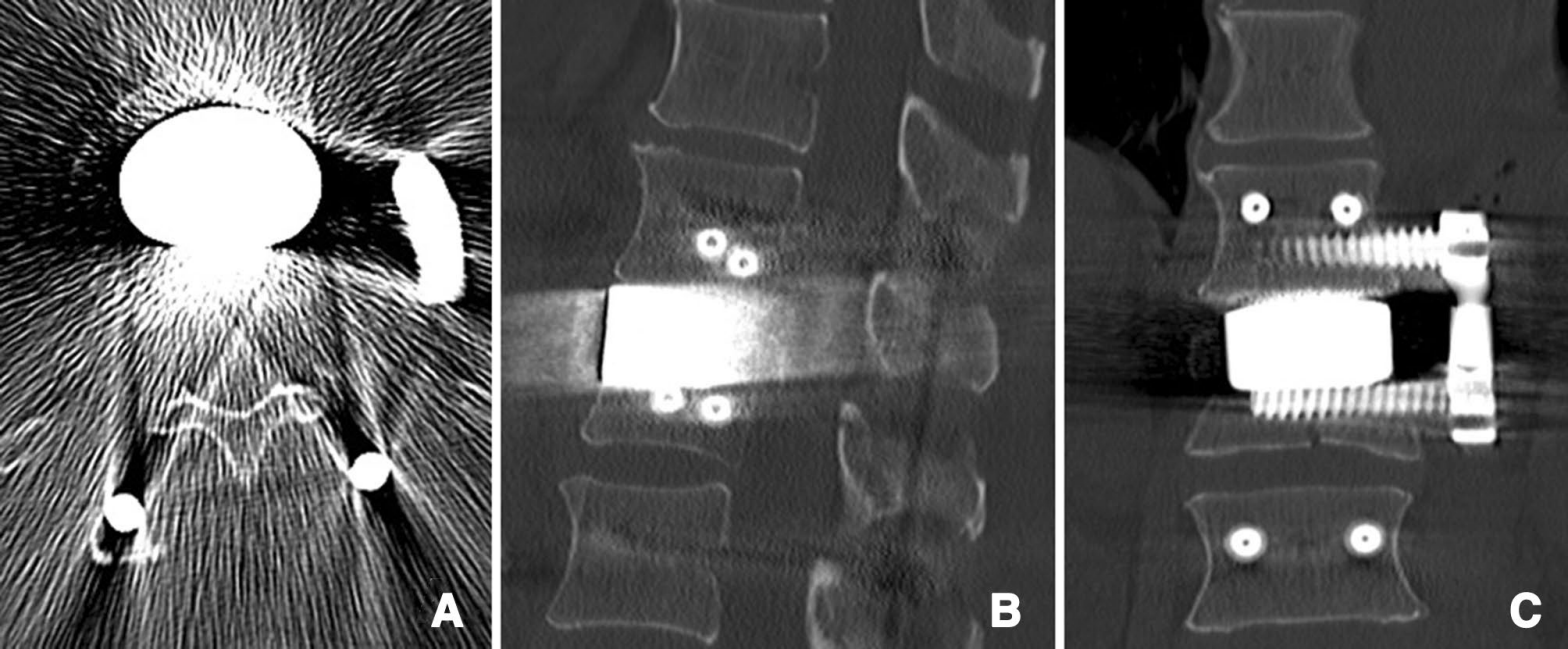
Postoperative CT of a 65-year-old man with a superior burst-split fracture of L1 (Magerl et al.^19^ 3.2.1, AOSpine A4^1^) treated by short segment posterior instrumentation and anterior one-level spondylodesis using a porous tantalum fusion implant (PTFI). Representative axial (**A**), sagittal (**B**) and coronal (**C**) slices.

Posterior reduction and short-segment fixation using angular stable pedicle screw systems (one level above and one level below the fracture level) were performed in all patients as the first step. In the second surgical step, reconstruction of the anterior spinal column was performed. For the T12 and L1 vertebral levels, fusion was performed using the video-assisted thoracoscopic approach described by Beisse^21^. Fractures of L2 to L4 were surgically treated via an anterolateral retroperitoneal approach (mini-lumbotomy). In otherwise healthy patients, we routinely perform combined anteroposterior stabilization as a one-stage procedure. For older trauma patients and patients with preexisting conditions, we prefer a two-stage procedure. In all cases, the intervertebral disc adjacent to the fractured end plate was removed by incising the anulus fibrosis and using a series of rongeurs and curettes. After discectomy, a bone sparing corporectomy (removal of the fractured superior vertebral body) was performed. Before corporectomy, the segmental vessels were identified, dissected free, and ligated. Afterwards, the length of the bony defect was measured. For reconstruction of the vertebral body, either an ICBG (Fig. 2) or a PTFI (trabecular metal, TM-400, Zimmer, Germany) (Fig. 3) was used. ICBG was harvested from the left iliac crest. The bone graft was then transplanted to the defect in a press-fit manner. After reconstruction, the affected segment was further stabilized with a minimally invasive plating system (Telefix, Synthes, Switzerland) in both groups. Prior to wound closure, intraoperative imaging was performed to check the adequacy of reduction, position, and length of screws and the overall coronal and sagittal spinal alignment. In both groups, adequate postoperative analgesia was provided.

### Radiological monitoring

Multislice computed tomography (CT), including sagittal and coronal reconstruction, was carried out pre- and postoperatively. In cases where a posterior tension band injury was suspected on CT, an additional MRI scan was performed. Lateral plain radiographs were taken in a standing position in the first and sixth weeks postoperatively and 3, 6, 9, 12, 18 and 24 months postoperatively. The injuries were classified according to both the comprehensive classification system of thoracic and lumbar injuries published by Magerl et al.^19^ and the AOSpine thoracolumbar spine injury classification system^1^. Sagittal spinal alignment was measured pre- and postoperatively as the mono- and bisegmental Cobb angles described by Reinhold et al.^22^. A kyphotic deformity was defined as a negative (−) angle, and a lordotic deformity was defined as a positive (+) angle. Before removal of the internal fixator (9–12 months postoperatively), a third CT scan was performed to accurately assess the state of interbody fusion. The states of fusion included complete fusion, partial fusion, and unipolar and bipolar nonfusion. Complete fusion was defined as the presence of at least three bony bridges or a single bony bridge wider than 3 mm between vertebral bodies.

### Exclusion of osteoporosis

All postmenopausal women in this study underwent quantitative CT (QCT) scans of the spine or DXA scans of the hip postoperatively. The fractured vertebrae were excluded from the assessment. The diagnostic thresholds of osteoporosis were defined as a T-score of -2.5 standard deviations (SDs) or more below the average value for young healthy women (DXA, WHO definition) or a bone mineral density of 80 mg/cm^3^ or below (QCT) in spinal trabecular bone^23^.

### CT based measurements of the ICBGs

**Figure 4:**
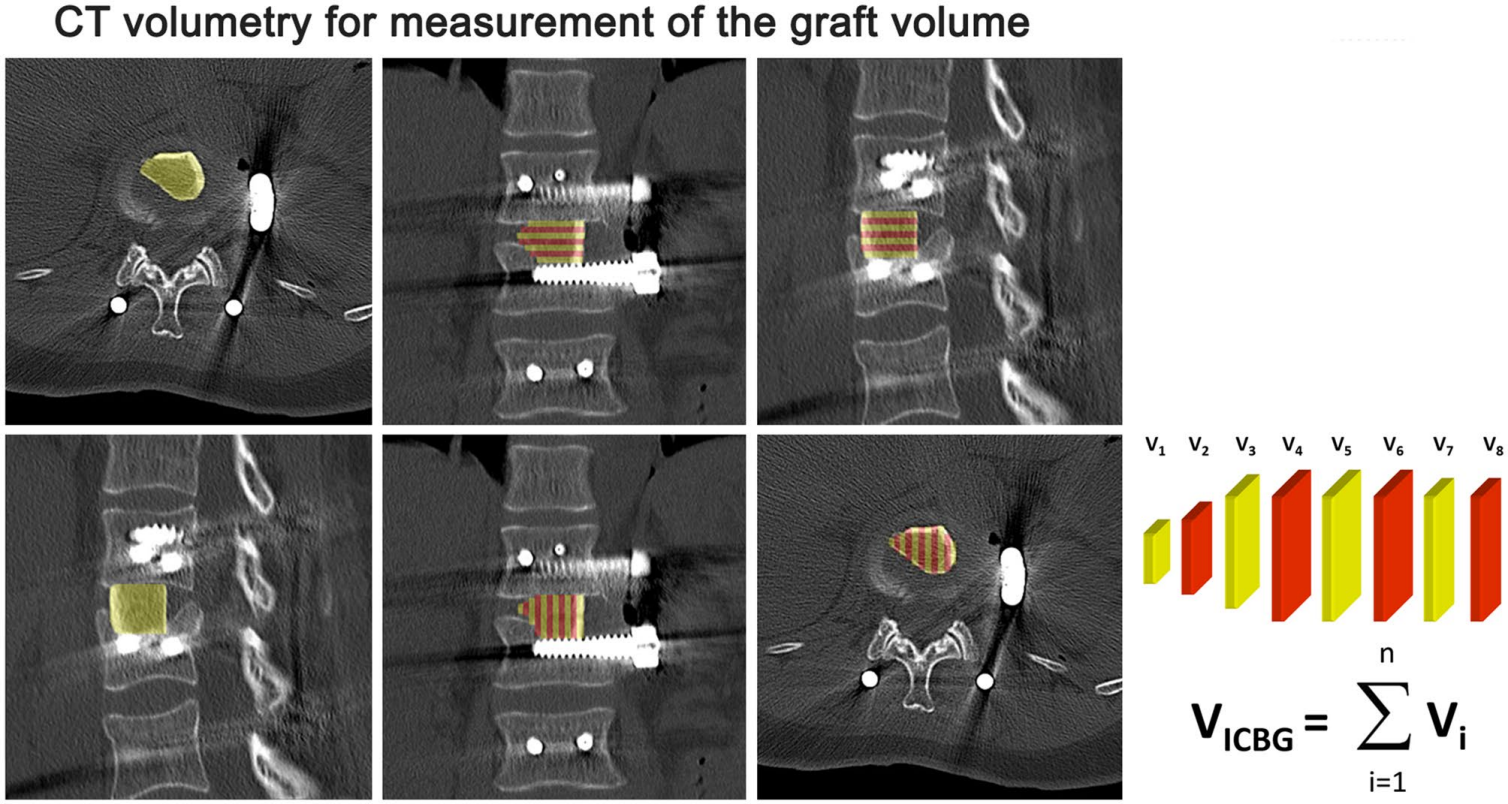
CT volumetry for estimation of the graft volume (ICBG). The CT scan demonstrates how the volume was measured in all three plains.

Postoperatively and before removal of the internal fixator (9–12 months postoperatively), axial CT scans and sagittal and coronal reconstructions were performed using a nominal slice thickness of 3 mm. The contours of the iliac crest graft (region of interest—ROI) were delineated in each single axial and sagittal slice using Syngo imaging software (Siemens, Germany). The iliac crest graft volume was determined by multiplying the area within each freehand contour by the effective slice thickness and summing over the total number of slices (Fig. 4). The measurement was carried out on both the axial and sagittal images. The average of the two measurements for each iliac crest graft was used for analysis. Furthermore, the length and average footprint of the grafts were determined postoperatively and before removal of the posterior hardware.

### Visual analog scale (VAS) spine score and Odom’s criteria

The VAS spine score (0–100 points) is a self-reported score used to measure subjective back function^24^. A higher VAS score represents less severe back problems. The VAS spine score was measured at admission to determine the status before surgery and at each scheduled follow-up visit (see follow-up). Moreover, the clinical outcome was considered excellent, good, satisfactory or poor based on Odom's criteria^25^ (Table 3).

**Table 3:** Odom’s criteria^25^.

Outcome	Description
Excellent	No complaints; able to carry out physical activities
Good	Intermittent discomfort, able to carry out physical activities
Satisfactory	Subjective improvement; physical activities significantly limited
Poor	Symptoms and signs unchanged or exacerbated

### Follow-up (FU)

FU visits after discharge from the hospital were scheduled 6 weeks and 3, 6, 9, 12, 18 and 24 months after surgery.

### Statistical analysis

The data were analyzed using SPSS (version 16, SPSS Inc., Chicago, IL, USA) for Windows. Standardized statistical tests [t-test, Wilcoxon’s test, Mann–Whitney test, multivariate data and regression analysis (ANOVA)] were used for comparisons. The data are presented as the mean ± SD. Significance was accepted at a probability value of less than or equal to 0.05.

### Ethical approval

The study was approved by the institutional review board and performed in accordance with the ethical principles originating from the Declaration of Helsinki and in compliance with Good Clinical Practice.

### Consent on participation

All patients gave their informed consent on participation of this study.

## Results

### Patient population

There were no significant differences in the baseline characteristics between the two groups with respect to age, sex, smoking behavior, duration of FU, VAS spine score before surgery or fracture level (Table 4). All patients suffered a high-energy traumatic event, such as a car accident or a fall from a height.

**Table 4:** Patient characteristics.

Patient characteristics	PTFI	ICBG	p-value
Sex ratio [males to females]	1.3: 1	1.1: 1	0.8
Age [Years]	42.6 ± 13.7	43.7 ± 12.9	0.8
Ratio of smokers to nonsmokers	1: 3.6	1: 3.2	0.9
Fractures within the thoracolumbar junction (T11-L2)	96% (22/23)	86% (18/21)	1
Load sharing classification score^3^	6.6 ± 0.5	6.5 ± 0.5	0.6
Follow up time	533 ± 262	487 ± 274	0.6

### Implant and initial transplant size

See Table 5.

**Table 5:** Height, depth, width and volume of PTFIs and ICBGs (initial measurement). The measurements are presented as the means ± standard deviations.

Measurement	PTFI	ICBG	p-value
height [mm]	15.7 ± 3.6	13.8 ± 2.4	0.05
depth [mm]	21 ± 0	11.0 ± 2.1	< 0.005
width [mm]	32 ± 0	29.6 ± 4.8	0.03
volume [ml]	10.5 ± 2.4	4.4 ± 1.2	< 0.005

### Exclusion of osteoporosis

None of the postmenopausal women fulfilled the criteria for osteoporosis.

### Comparison of the surgical time and length of hospital stay

No significant differences in the length of hospitalization (PTFI: 15.1 ± 7.5 days; ICBG: 16.1 ± 4.3 days) or surgical time (PTFI: 133.8 ± 23.2 min; ICBG: 142.4 ± 21.7 min) were observed between the groups. However, in the implant group, there was a clear trend showing a reduction in operative time with increased experience (learning curve).

### VAS spine score

**Figure 5:**
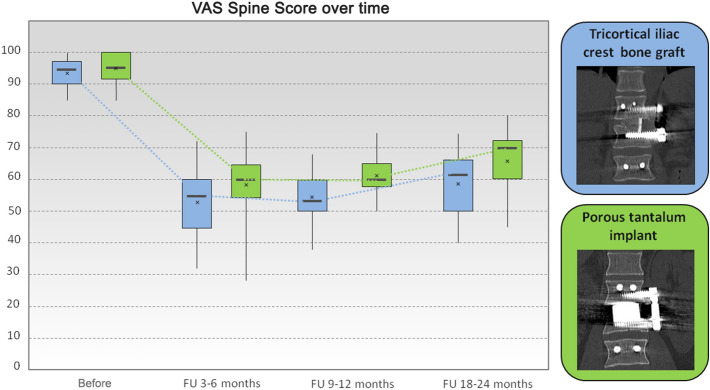
Box and whisker plot of the VAS spine score at all time points. For each box, the lower border is the 25th percentile, and the top border is the 75th percentile. The dark line in the middle of the box is the 50th percentile (the median), and whiskers denote the 2.5th and 97.5th percentiles. The cross indicates the mean value.

The average initial VAS spine score, representing the status before surgery, was 93.9 ± 4.8 points (ICBG group 93.5 ± 4.6; PTFI group 94.4 ± 5.1). The average VAS spine score at 18 to 24 months postoperatively was 62.9 ± 10.5 points (ICBG group 58.8 ± 11.0; PTFI group 66.5 ± 8.7) (Fig. 5). The difference between the two groups at the time of the last FU was not statistically significant.

### Odom’s scale

Nine to 12 months after surgery, 10 of the 44 (23%) patients were asymptomatic (Odom I), and 26 (59%) had only mild symptoms and normal ability to perform daily activities (Odom II). Eight (18%) patients showed satisfactory results. Regarding Odom's criteria, the results were not significantly different between the groups.

### Radiological findings

**Figure 6:**
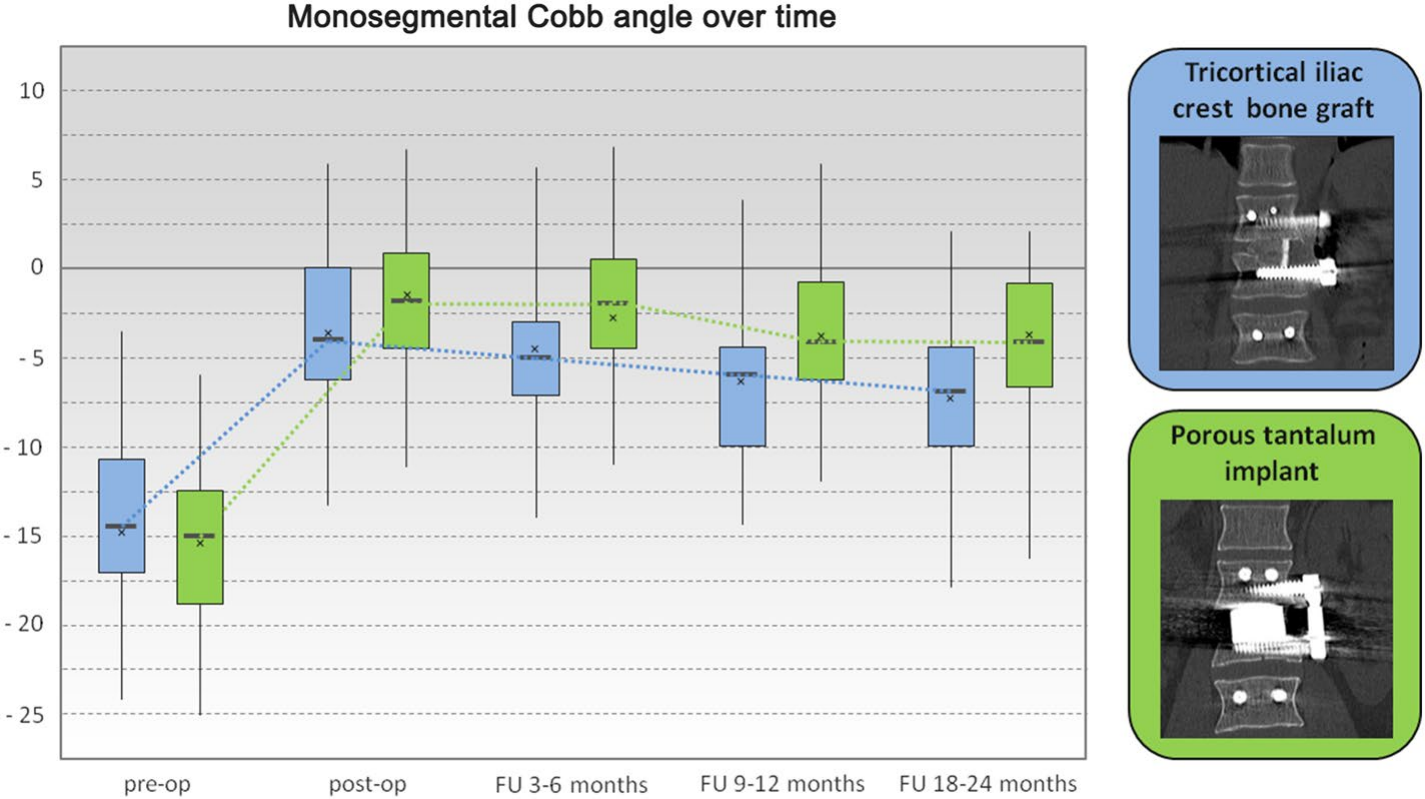
Box and whisker plot showing the changes in the monosegmental Cobb angle. For each box, the lower border is the 25th percentile, and the top border is the 75th percentile. The dark line in the middle of the box is the 50th percentile (the median), and whiskers denote the 2.5th and 97.5th percentiles. The cross indicates the mean value.

At the time of surgery, the severity of kyphotic deformity (monosegmental Cobb angle) varied according to the level of injury, with an average angle of − 15.5 ± 5.5° (ICBG group − 14.8 ± 5.8°; PTFI group − 16.2 ± 5.2°). Postoperatively, this angle was − 2.7 ± 4.9° (ICBG group − 3.6 ± 5.4°; PTFI group − 1.9 ± 4.3°), showing a significant correction of 12.8 ± 4.5° (range 4–21°; *p* < 0.005) (ICBG group 11.1 ± 3.9°; PTFI group 14.3 ± 4.6°). At the time of last FU, the monosegmental Cobb angle showed a slight loss of correction of 2.5 ± 2.6° (ICBG group 3.5 ± 3.1°; PTFI group 1.6 ± 1.7°), but the difference between the groups was not statistically significant. However, the magnitude of improvement in the deformity from pre- to postoperatively differed significantly between the two groups. The change in the monosegmental Cobb angle from preoperatively to the last FU at 18–24 months after surgery was 7.8 ± 4.5° for the iliac crest graft patients and 12.3 ± 5.1° for the tantalum implant group (*p* < 0.005) (Fig. 6).

### Radiographic evaluation of the state of interbody fusion

**Figure 7:**
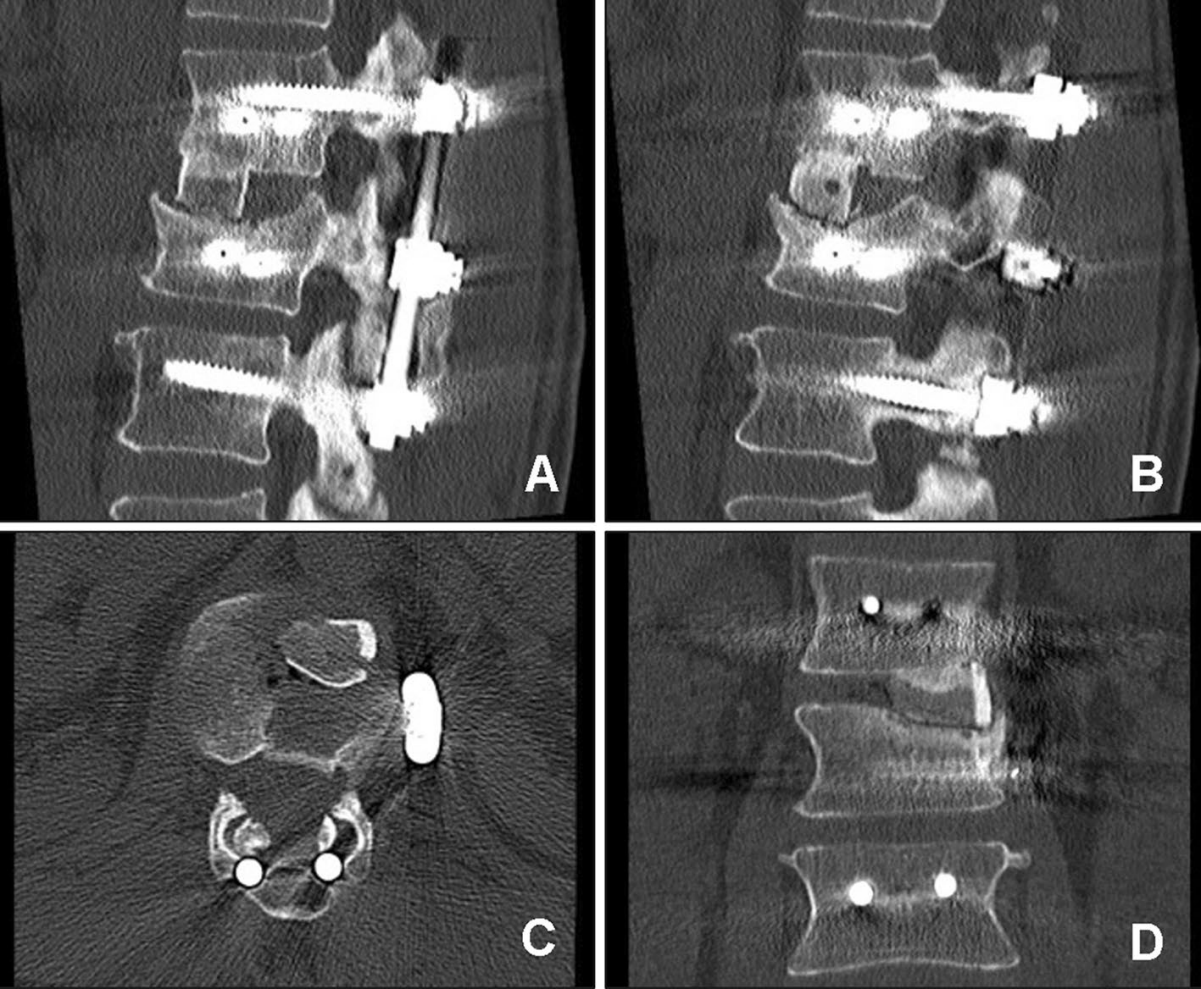
CT of a 41-year-old man (superior incomplete burst fracture of L2, Magerl et al.^19^ A3.1.1, AOSpine A3^1^) 11 months after short segment posterior instrumentation and anterior one-level spondylodesis using an ICBG, showing unipolar nonfusion between the lower part of the iliac crest bone graft and the adjacent vertebrae (L3).

There were two failed fusions in the ICBG group: a unipolar (Fig. 7) and a bipolar nonfusion. Tantalum, however, causes relevant beam hardening artifacts in CT scans that limit the diagnostic information of any FU imaging examinations (Fig. 3A). No direct (gap between implant and bone) or indirect radiological signs (e.g., implant-induced osteolysis) of failed fusion were detectable in the PTFI group.

### Complications and reoperation

**Figure 8:**
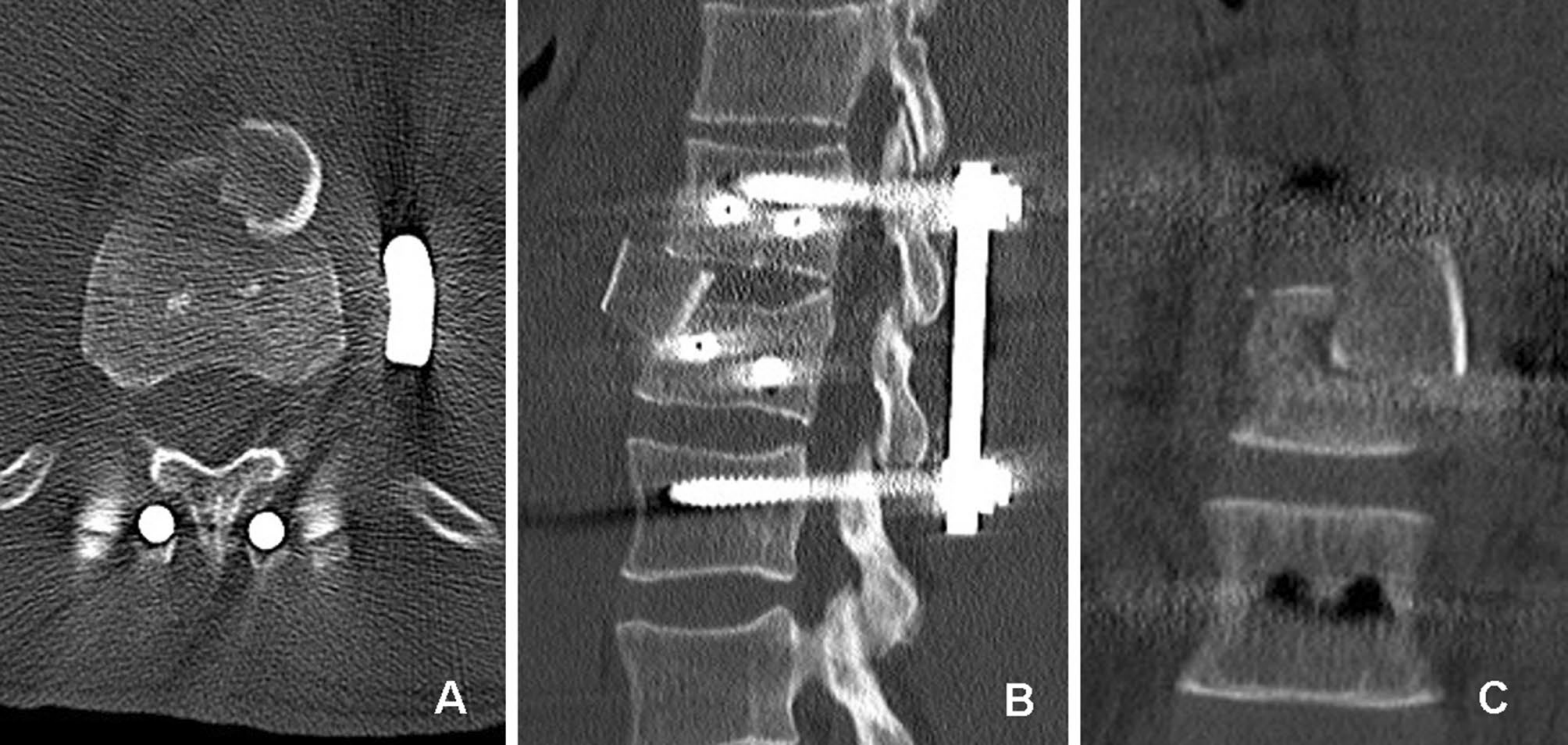
Postoperative CT of the thoracolumbar junction showing a dislocation of the iliac crest bone graft, which was caused by the caudal-anterior screw of the plating system.

Only one patient in the ICBG group required revision surgery because of intraoperative graft dislocation (Fig. 8). Implant loosening or breakage was not observed. There was no donor site complication in the ICBG group.

### CT based measurements of ICBGs

The volume and length of the ICBG were measured immediately and after 9–12 months using CT. The autografts lost an average of 12% (482 mm^3^) of their initial volume of 4453 ± 1327 mm^3^ and 9% (1.3 mm) of their height (13.9 ± 2.8 mm) (Table 5).

## Discussion

Burst fractures of the thoracolumbar spine may cause instability and deformity of the spine as well as neurologic damage^26^. Therefore, the goals of surgical intervention are decompression of the neural elements, restoration of the vertebral body, correction of the angular deformity, and spinal stabilization. In fact, the use of posterior instrumentation alone for the treatment of severe thoracolumbar burst fractures presents a high risk of failure, instability, or loss of correction^3–5^. McCormack et al.^3^ proposed the load sharing classification system to help identify fractures that require additional anterior stabilization. With this system, the fractured vertebra is scored on the basis of the amount of comminution, fracture apposition, and kyphosis. Fractures with a score of 7 or more points require anterior stabilization, whereas patients with a score of 6 or fewer points can be treated with an isolated posterior approach. Among the 28 patients studied by McCormack et al.^3^, all the patients with a score of 7 or more points (10 out of 28) had breakage of the implant.

The anterior approach provides excellent decompression and vertebral column reconstruction. However, when used alone, it may not resist additional destructive flexion forces when the posterior ligaments have been rendered incompetent^27^. The use of a combined anterior and posterior technique provides ideal restoration of vertebral body height and rigid fixation in all 3 planes of spinal motion. On the other hand, posterior-anterior stabilization is technically more difficult, is more costly and potentially leads to more morbidities than posterior-only stabilization^12^. However, according to Schnake et al.^28^, additional anterior fusion with modern implants may be a safe procedure that leads to a satisfactory long-term radiological and clinical outcomes.

Today, there are several options available for achieving interbody fusion and physiological anterior column support, including autografts, allografts and metallic fusion devices. However, the optimum method for reconstruction of the anterior column in thoracolumbar burst fractures remains controversial. For one-level fusion, autogenous tricortical bone grafts from the iliac crest remain the gold standard against which all other fusion devices must be compared. The aim of this prospective observational study was to compare two different techniques of performing anterior one-level fusion for thoracolumbar burst fractures using either an ICBG or an implant made of porous tantalum to support the anterior column.

The ideal bone graft material should provide osteogenesis, osteoinductivity, osteoconductivity, and osseointegration. Among all types of bone grafts, only autografts possess all these features. The iliac crest is an easily accessible donor site for autologous bone, but severe morbidities, e.g., persistent pain and discomfort at the donor site, have been widely reported in the literature^13,16^. Moreover, rare but severe types of complications are possible, such as damage to the superior gluteal artery, fourth lumbar artery, iliolumbar artery, and deep circumflex iliac artery; damage to the femoral nerve and ilio-inguinal nerves; ureteral injuries; gastrointestinal hernias; ileus; hematomas; pelvic instability; and fractures^29^. The fusion rates reported in the literature vary widely, ranging from 66 to 97%^14,30^. In the current study, the level of osseous integration of the autogenous transplants was measured by CT at 9 to 12 months after surgery (before hardware removal), revealing a fusion rate of 90% (19/21) for the ICBG group. The tantalum implant used in the other group caused significant artifacts. These artifacts severely degraded the quality of CT images within this group, making them diagnostically unusable. For patients with anterior one-level fusion using a PTFI, plane radiography might have higher diagnostic accuracy in the assessment of spinal fusion. Neither CT nor plane radiography showed direct or indirect signs of failed fusion.

Negative effects of nicotine on spinal fusion have been reported^31^, but no differences in smoking prevalence between the two groups were observed in the current study.

Recent reports have indicated that the surgical time can be reduced by using a fusion device instead of taking the time to harvest an autograft^32^. However, the current study showed no significant reduction in surgical time using a PTFI. Nevertheless, a trend showing a decreased operative time as the number of procedures performed by the surgeon increased was noted. The mean operative time in the PTFI group might have been influenced by the learning curve phenomenon. After the learning curve is overcome, the use of a PTFI in anterior one-level fusion may shorten the operative time.

When the combination of dorsal and ventral stabilization was used in the thoracolumbar burst fractures, significant restoration of the sagittal profile was observed. The monosegmental Cobb angles at 9–12 months after surgery and at the time of last FU were significantly different compared to those measured preoperatively. In both groups, a slight but statistically nonsignificant loss of correction over time was observed. Significantly better restoration of the sagittal profile was obtained with the PTFI than with the iliac bone graft at the long-term FU.

We assume that when a PTFI is used, better primary restoration of the sagittal profile can be obtained because the implant allows very precise restoration of the defect using intraoperative probes, which can be adapted to the patient's defect in intervals of millimeters after partial corporectomy and dissection of the intervertebral disc. When a slightly overdimensioned implant is used, impaction of the cancellous bone of the affected vertebra and further correction of the sagittal profile can be obtained. When an autologous ICBG is used, decrease in volume and height were observed over time, especially when no additional ventral stabilization was performed. In those cases, the autologous iliac bone graft lost 40% of its initial volume and 25% of its initial height, which was linked to a significant loss of correction over time^33^. However, even under the mechanical cover of an additional implant, we observed a loss of volume (9%) and height (12%) in the ICBG group, and no loss of volume or height was observed in the PTFI group.

We further assume that the PTFI provides greater mechanical strength, as its cross-sectional area and bearing surface are significantly larger than those of the ICBG (500 mm^2^ vs. 150 mm^2^). The cortical bone of iliac crest grafts is dense, and their fusion surface (footprint) is small^14^. A large footprint affords substantial biomechanical advantages^34^. The subsidence of implants and loss of correction can be reduced^22^.

We assume that the sum of these effects (better initial restoration of the sagittal profile and smaller losses of volume and height) in the PTFI group led to a significantly better mid-term radiological result. However, a significant difference in the VAS score was not observed, but no donor site morbidities or implant-specific complications were observed, so we think using a PTFI is equivalent to using a ICBG for anterior spondylodesis in the treatment of burst fractures of the thoracolumbar spine.

While the focus of this study lies on combined posterior-anterior approaches, it must be acknowledged that a posterior-only approach is another safe procedure to achieve decompression and stable reconstruction. Advantages of this less invasive surgery include a shorter operation time, less blood loss, and fewer complications. However, the long-term influence of this approach on the sagittal spine profile remains unclear. Despite this, a posterior-only approach is a valuable alternative. Disadvantages of a combined posterior-anterior approach are higher costs and potentially higher morbidity compared to posterior-only stabilization. In patient collectives with a high surgical risk profile, such as the elderly, it is therefore reasonable to apply a posterior-only approach to stabilize the spine and fix several motion segments. In the younger population, combined posterior-anterior approaches may be more advantageous in maintenance of the long-term correction^12,35^.

A limitation of our study was the selection criteria by surgeons preference what can cause potential bias. All involved surgeons used PTFI as well as ICBG for the spondylodesis.

## Conclusion

The authors conclude that both techniques are effective and safe for treating unstable thoracolumbar injuries. The current study shows advantages for porous tantalum implants. In particular, significantly better medium-term radiological results were detected. The authors suggest that porous tantalum fusion implants are a good alternative to autologous bone grafting, as they prevent complications associated with harvesting. However, additional randomized controlled studies are still required to determine the efficacy of this new method in the treatment of thoracolumbar burst fractures.

## Data Availability

Raw data is available on request. Contact: heintel_t@ukw.de.
